# No association between dietary patterns and depressive symptoms among a community-dwelling population in Japan

**DOI:** 10.1186/1744-859X-11-24

**Published:** 2012-09-24

**Authors:** Norio Sugawara, Norio Yasui-Furukori, Shoko Tsuchimine, Ayako Kaneda, Koji Tsuruga, Kaori Iwane, Noriyuki Okubo, Ippei Takahashi, Sunao Kaneko

**Affiliations:** 1 Department of Neuropsychiatry, Hirosaki University School of Medicine, Hirosaki, Japan; 2 Department of Social Medicine, Hirosaki University School of Medicine, Hirosaki, Japan

## Abstract

**Background:**

Studies of the associations between diet and depression have primarily focused on single nutrients or foods. Recently, dietary patterns representing a combination of foods have attracted more interest than individual nutrient. The objective of this study was to examine the association between dietary patterns and depressive symptoms among a community-dwelling population in Japan.

**Methods:**

We examined the association between dietary patterns and the risk of depression among 791 Japanese community-dwelling individuals. Diet was assessed with a validated brief-type self-administered diet history questionnaire (BDHQ). Dietary patterns from 52 predefined food groups [energy-adjusted food (g/d)] were extracted by principal component analysis. The Center for Epidemiologic Studies Depression Scale (CES-D) with a cut-off point of 16 was used to assess the prevalence of depression.

**Results:**

A total of 97 subjects (12.3%) were classified as having depression. Four dietary patterns were identified: “Healthy”, “Western”, “Bread and confectionery”, and “Alcohol and accompanying” dietary patterns. After adjusting for potential confounders, the dietary patterns were not related to the risk of depression.

**Conclusions:**

The present study failed to find associations between dietary patterns and the risk of depression. However, the interpretation of our results was hampered by the lack of certain data, including employment physical activity and longitudinal observations. Potential associations between dietary patterns and depressive symptoms were not completely ruled out. Future research exploring dietary patterns and depressive symptoms is warranted.

## Background

Depression is a serious affective illness with a high lifetime prevalence rate [1]. The World Health Organization has estimated that major depressive disorder will become the second most important factor in worldwide disability-adjusted life-years (DALYs) by the year 2020 [2]. However, the unsatisfactory results of pharmacotherapy and high prevalence of somatic symptoms and physical illness in patients with depression imply that the serotonin hypothesis is not a sufficient approach to the aetiology of depression [3]. As the social and economic costs of depression continue to increase, it is essential to find alternative therapeutic solutions [4].

Diet has been suggested as one modifiable factor that plays a role in depression [5]. Previous studies [6-8] have examined the association between depressive symptoms and specific nutrients, such as folate, other B vitamins and n-3 polyunsaturated fatty acids (PUFAs), on the basis of their role in biosynthesis, metabolism and neuronal membrane stability. However, the results have not been consistent. These nutrients are consumed in dietary combinations, and they may have interactive or synergistic effects that make studying them in isolation difficult [9]. Dietary patterns that represent a combination of foods may be more strongly associated with disease risk than individual foods or nutrients [10-12].

The hypothesis that dietary patterns may be associated with depression risk has been suggested. Some studies conducted in Western populations have supported this hypothesis [13-15]. However, two studies of Japan [16,17], which has different dietary patterns from Western nations, have found contradictory results.

In the present study, we examined the association between dietary patterns and depressive symptoms among a community-dwelling population in Japan. We hypothesised that dietary patterns consisting of high intakes of vegetables and fish would be associated with decreased risk of depression.

## Methods

### Participants

The subjects were 791 volunteers (22–86 years old, 303 males and 488 females) who participated in the Iwaki Health Promotion Project 2011. They were residents of Iwaki district, a rural area of Hirosaki city, in northern Japan. Iwaki district is a stable community with a population 11,863. The data collection for this study was approved by the Ethics Committee of the Hirosaki University School of Medicine, and all of the subjects provided written informed consent before participating in the project. The demographic data (age, sex, level of education, and marital status), life style (smoking, drinking), having exercise habits (defined as 4 and more times per week), and medical history were obtained from self-reported questionnaires and interviews. The subject’s height and weight were measured, and the body mass index (BMI) was calculated.

### Procedure

The Japanese version of the Center for Epidemiologic Studies Depression Scale (CES-D) [18,19] was administered to all of the participants to measure their depressive symptoms. The CES-D is a 20-item self-report measure that focuses on depressive symptoms during the week prior to administering the questionnaire. The maximum score on this scale is 60, and depression was considered to be present when a subject had a CES-D scale of 16 or more.

Dietary habits during the preceding month were assessed using a validated brief self-administered diet history questionnaire (BDHQ) [20] that contained queries about the consumption frequency of 56 foods and beverages and 9 dishes commonly consumed in general Japanese populations. The dietary intakes of energy and selected nutrients were estimated using an *ad hoc* computer algorithm for the 56 foods and beverages of the BDHQ and the Standard Tables of Food Composition in Japan [21,22]. The validation study of the BDHQ used 16-day dietary records as a reference in 92 men and 92 women, aged 31–76 years, and found median (interquartile range) Pearson correlation coefficients for 42 nutrients of 0.56 (0.41 to 0.63) for the men and 0.54 (0.45 to 0.61) for the women [23].

### Statistical analysis

We derived dietary patterns through a principal component analysis of the energy-adjusted intakes that used a density method for the 52 food and beverage items (excluding 4 items that overlapped with others). We used eigen values, the scree test, and the interpretability of the factors to determine the number of factors to retain. The factors each had eigen values greater than one. The scree plots dropped substantially after the fourth factor (from 2.19 to 1.68) and remained similar after the fifth factor (1.68 for the fifth and 1.61 for the sixth factor); thus, we decided to retain four factors. The factor scores for each dietary pattern and for each individual were calculated by summing the food item intakes weighted by their factor loadings. The factor scores were categorised into tertiles.

The Student's unpaired t test (for the continuous variables) or Chi-square test (for categorical variables) was used to compare the subjects with and without probable depression. Trend associations across the tertile categories of each dietary pattern were assessed using the Cochran-Armitage trend test for the categorical variables and linear regression analysis for the continuous variables, with ordinal values from 0 to 2 assigned to the tertile categories of each dietary pattern.

A logistic regression analysis was used to assess the relationship between dietary patterns and depressive symptoms. The minimally adjusted model was adjusted for age, gender and exercise habits, and the fully adjusted model was further adjusted for body mass index, education, marital status, current smoking, history of hypertension and diabetes mellitus. A p < 0.05 result was considered to be statistically significant. The data were analysed using the PASW Statistics PC software for Windows, Version 18.0.0 (SPSS Inc., Chicago, IL, USA) and R 2.10.1 [24]. R 2.10.1 was used for the Cochran-Armitage trend test only.

## Results

The characteristics of the study subjects according to their depressive symptoms are shown in Table 1. Ninety-seven subjects (12.3%) were identified as having probable depression (CES-D scale scores of > 16). There were no significant differences between the subjects with and without depressive symptoms.

**Table 1:** Characteristics according to subjects with and without depressive symptoms

	** Subjects with**	** Subjects without**	**p value**^**c**^
	**depressive symptoms**^**a**^	**depressive symptoms**^**b**^	
Number of subjects	97	694	
Age	57.0 ± 14.1	57.6 ± 13.6	0.673
BMI	22.9 ± 3.7	23.1 ± 3.5	0.756
Amount of education (year)	11.5 ± 2.1	11.5 ± 2.1	0.757
Male (%)	34.0	38.9	0.354
Married (%)	74.2	74.2	0.997
Current smoking (%)	13.4	15.6	0.580
Habitual alcohol intake (%)	43.3	44.7	0.799
Exercise habit^d^ (%)	11.3	12.8	0.680
History of hypertension (%)	29.9	30.8	0.851
History of diabetes mellitus (%)	5.2	5.9	0.767
Dietary intake			
Energy (kcal)	1870 ± 639	1942 ± 609	0.279
Folate (ug/1000kcal)	178 ± 73	173 ± 62	0.431
Riboflavin (Vitamin B2) (mg/1000kcal)	0.67 ± 0.19	0.66 ± 0.18	0.923
Pyridoxine (Vitamin B6) (mg/1000kcal)	0.65 ± 0.18	0.65 ± 0.17	0.887
Cobalamin (Vitamin B12) (μg/1000kcal)	5.2 ± 3.0	5.1 ± 2.7	0.809
n-3 polyunsaturated fatty acids (g/1000kcal)	1.43 ± 0.50	1.44 ± 0.50	0.866

Abbreviation: *BMI*, body mass index.

a Subiects wth a Center for Epidemiologic Studies Depresion scale score≧16.

b Subiects wlh a Center for Epidemiologic Studies Depresion scale score <16.

c For continuous variables non-paired t-test was used; for categorical variables, chi square test was used.

d Exercise habit was defined as 4 and more times per week.

We identified four dietary patterns by principal component analysis (Table 2). The first factor, which loaded heavily on vegetables, seaweeds, tofu, fruits, and fish, was labelled the “healthy” dietary pattern. The second factor, which had high loadings for beef/pork, processed meat, mayonnaise/dressing, ice cream, bread, spaghetti and macaroni, was labelled the “Western” dietary pattern. The third factor represented high intakes of confectioneries and bread and low intakes of vegetables; this pattern was termed the “bread and confectionery” dietary pattern. The fourth factor was characterised by high intakes of noodles, squid/octopus/shrimp/shellfish, and alcoholic beverages; thus, it was termed the “alcohol and accompanying” dietary pattern. These four dietary patterns accounted for 10.5, 4.6, 4.4 and 4.2, respectively, of the variance in food intakes and explained 23.7% of the variance.

**Table 2:** **Factor loading matrix for major dietary patterns identified by principal component analysis**^**a**^

	**Healthy dietary pattern**	**Western dietary pattern**	**Bread and confectionery dietary pattern**	**Alcohol and accompanying dietary pattern**
Milk and yogurt	0.188	-	-	-
Chicken	-	0.284	-	-
Pork/beef	-	0.455	−0.189	-
Ham/sausage/bacon	-	0.480	−0.187	-
Liver	-	-	-	0.183
Squid/octopus/shrimp/shellfish	-	-	-	0.380
Small fish with bones	0.219	−0.230	-	0.180
Canned tuna	0.193	-	-	-
Dried fish/salted fish	0.295	−0.197	-	-
Oily fish	0.308	−0.295	-	0.206
Lean fish	0.362	−0.238	-	0.216
Egg	-	0.191	−0.257	-
Tofu/atsuage^b^	0.516	-	-	-
Natto^c^	0.268	−0.190	-	-
Potatoes	0.475	-	-	-
Pickled green leaves vegetables	0.272	−0.280	-	-
Other pickled vegetables	-	−0.340	0.158	-
Lettuces/cabbage(raw)	0.508	0.219	−0.196	-
Green leaves vegetables	0.623	-	−0.186	-
Cabbage/Chinese cabbage	0.651	-	−0.259	-
Carrots/pumpkin	0.682	-	−0.199	-
Japanese radish/turnip	0.559	-	-	-
Other root vegetables	0.636	-	−0.173	-
Tomatoes	0.447	-	-	-
Mushrooms	0.622	-	−0.156	-
Seaweeds	0.533	-	−0.164	-
Western-type confectionneries	-	0.227	0.501	−0.325
Japanese-type confectioneries	-	-	0.505	−0.226
Rice crackers/rice cake/okonomiyaki	-	-	0.568	−0.164
Ice cream	-	0.364	0.239	-
Citrus fruit	0.321	-	0.231	0.190
Persimmons/strawberries/kiwifruit	0.419	-	-	-
Other fruit	0.332	-	0.266	-
Mayonnaise/dressing	0.246	0.398	-	-
Bread	-	0.344	0.320	−0.153
Buckwheat noodles	-	-	0.185	0.488
Japanese wheat noodles	-	-	0.305	0.450
Chinese noodles	−0.313	-	-	0.442
Spaghetti and macaroni	-	0.326	-	0.226
Green tea	0.265	-	-	-
Black tea/oolong tea	-	0.224	-	-
Coffee	-	0.281	−0.166	-
Cola drink/soft drink	−0.230	0.218	-	0.168
100% fruit and vegetable juice	-	-	0.174	0.188
Rice	−0.320	−0.488	−0.291	−0.533
Miso soup	-	−0.363	−0.243	−0.270
Sake	−0.308	−0.155	−0.213	0.277
Beer	−0.308	-	−0.334	0.355
Shochu	−0.352	-	−0.289	0.340
Wine	-	-	-	0.204

a Factor loading less than ±0.15 represented by a dash for simplicity. Omitted in the table were food items with factor loadings less than ±0.15 for all dietary patterns (Reduced fat milk and yogurt, Whisky).

b Deep fried tofu.

c Fermented soybeans.

Table 3 shows the characteristics according to tertiles of the dietary pattern scores. The subjects with higher scores for the “healthy” dietary pattern were more likely to exercise and less likely to be males, habitual drinker and current smokers. The healthy pattern was positively associated with age and intake of folate, riboflavin, pyridoxine, cobalamin, and n-3 PUFAs and was inversely associated with total energy consumption.

**Table 3:** Characteristics according to tertile categories of dietary pattern scores

	**Healthy dietary pattern**				**Western dietary pattern**				**Bread and confectionery dietary pattern**				**Alcohol and accompanying dietary pattern**			
	**Low tertile**	**Middle tertile**	**High tertile**	**Trend pa**	**Low tertile**	**Middle tertile**	**High tertile**	**Trend pa**	**Low tertile**	**Middle tertile**	**High tertile**	**Trend pa**	**Low tertile**	**Middle tertile**	**High tertile**	**Trend pa**
Number of subjects	264	263	264		263	265	263		263	265	263		264	264	263	
Age	54.0 ± 13.9	57.5 ± 13.8	61.1 ± 12.1	<0.001	64.4 ± 11.6	58.3 ± 11.9	49.8 ± 13.2	<0.001	53.5 ± 12.9	57.8 ± 13.4	61.3 ± 13.4	<0.001	55.9 ± 14.2	57.2 ± 13.8	59.5 ± 12.5	<0.01
BMI	23.2 ± 3.4	23.3 ± 3.6	22.7 ± 3.5	0.132	23.5 ± 3.6	23.2 ± 3.5	22.5 ± 3.4	<0.01	23.4 ± 3.7	23.0 ± 3.8	22.7 ± 3.1	<0.05	22.9 ± 3.8	22.8 ± 3.8	23.4 ± 3.0	0.08
Amount of education (year)	11.6 ± 2.1	11.4 ± 2.0	11.6 ± 2.1	0.850	10.9 ± 2.2	11.6 ± 2.0	12.2 ± 1.8	<0.001	11.9 ± 2.0	11.5 ± 2.0	11.2 ± 2.2	<0.001	11.4 ± 2.2	11.8 ± 2.1	11.4 ± 2.0	0.88
Male (%)	66.3	34.6	14.0	<0.001	42.2	38.1	34.6	0.07	48.7	37.4	28.9	<0.001	26.9	33.3	54.8	<0.001
Married (%)	72.3	77.9	72.3	1.000	70.0	72.8	79.8	<0.01	77.9	77.7	66.9	<0.01	71.6	71.6	79.5	<0.05
Current smoking (%)	29.5	12.2	4.2	<0.001	10.3	15.1	20.5	<0.01	22.4	12.8	10.6	<0.001	10.2	15.9	19.8	<0.01
Habitual alcohol intake (%)	65.2	43.7	24.6	<0.001	43.0	49.1	41.4	0.73	64.6	43.4	25.5	<0.001	29.5	41.7	62.4	<0.001
Exercise habitb (%)	7.6	10.3	20.1	<0.001	18.3	11.7	8.0	<0.001	11.0	12.8	14.1	0.29	6.8	12.1	19.0	<0.001
History of hypertension (%)	28.8	30.4	33	0.299	44.1	31.7	16.3	<0.001	44.1	31.7	33.1	0.16	23.9	32.2	36.1	<0.01
History of diabetes mellitus (%)	4.9	6.1	6.4	0.457	8.7	6.4	2.3	<0.01	5.3	4.2	8.0	0.19	4.9	6.1	6.5	0.450
Dietary intake																
Energy (kcal)	2047 ± 638	1967 ± 629	1784 ± 539	<0.001	1923 ± 604	1949 ± 611	1926 ± 626	0.96	1868 ± 608	1950 ± 592	1981 ± 635	<0.05	1773 ± 555	1920 ± 552	2106 ± 680	<0.001
Folate (μg/1000kcal)	122 ± 32	164 ± 31	234 ± 61	<0.001	176 ± 66	174 ± 63	170 ± 62	0.25	183 ± 79	171 ± 58	170 ± 50	<0.01	167 ± 71	176 ± 60	176 ± 60	0.1
Riboflavin (Vitamin B2) (mg/1000kcal)	0.52 ± 0.13	0.66 ± 0.12	0.81 ± 0.16	<0.001	0.65 ± 0.20	0.66 ± 0.17	0.60 ± 0.17	0.08	0.67 ± 0.20	0.66 ± 0.18	0.66 ± 0.16	0.55	0.64 ± 0.18	0.67 ± 0.17	0.68 ± 0.20	<0.01
Pyridoxine (Vitamin B6) (mg/1000kcal)	0.51 ± 0.11	0.63 ± 0.10	0.81 ± 0.14	<0.001	0.66 ± 0.18	0.65 ± 0.17	0.64 ± 0.16	0.33	0.67 ± 0.18	0.64 ± 0.18	0.63 ± 0.15	<0.01	0.59 ± 0.16	0.65 ± 0.16	0.70 ± 0.18	<0.001
Cobalamin (Vitamin B12) (μg/1000kcal)	3.7 ± 1.9	5.1 ± 2.1	6.6 ± 3.3	<0.001	5.8 ± 3.3	5.2 ± 2.6	4.4 ± 2.1	<0.001	4.7 ± 2.4	5.3 ± 3.0	5.4 ± 2.8	<0.01	4.0 ± 1.9	5.2 ± 2.4	6.2 ± 3.3	<0.001
n-3 polyunsaturated fatty acids (g/1000kcal)	1.11 ± 0.35	1.44 ± 0.37	1.77 ± 0.51	<0.001	1.47 ± 0.57	1.44 ± 0.48	1.41 ± 0.42	0.24	1.37 ± 0.50	1.47 ± 0.52	1.48 ± 0.47	<0.05	1.28 ± 0.36	1.48 ± 0.48	1.57 ± 0.58	<0.001

Abbreviation: *BMI*, body mass index.

a On the basis of the Cochran-Armitage trend test for categorical variables and linear regression analysis for continuous variables, assigning ordinal numbers 0-2 to tertile categories of each dietary pattern.

b Exercise habit was defined as 4 and more times per week.

The subjects with higher scores for the “Western” dietary pattern were more likely to be married and current smokers but less likely to exercise and have histories of hypertension and diabetes mellitus. The Western pattern was positively associated with education level and inversely associated with age, BMI, and cobalamin.

The subjects with higher scores for the “bread and confectionery” dietary pattern were less likely to be male, married, habitual drinker and current smokers. The bread and confectionery pattern was positively associated with age and intake of total energy, cobalamin, and n-3 PUFAs and was inversely associated with BMI, education level and intake of folate and pyridoxine.

The subjects with higher scores for the “alcohol and accompanying” dietary pattern were more likely to be male, married, habitual drinker and current smokers and to exercise and have a history of hypertension. The alcohol and accompanying pattern was positively associated with age and intake of total energy, riboflavin, pyridoxine, cobalamin, and n-3 PUFAs.

The odds ratios for depressive symptoms according to the tertile categories of each dietary pattern score are shown in Table 4. In both the minimally and fully adjusted model, no dietary pattern quartiles were associated with significantly increased or decreased risk of depressive symptoms.

**Table 4:** Odds ratio and 95%CIs for depressive symptoms according to tertiles of dietary pattern scores

	**No of cases**	**Minimally adjusted model**	**p value**	**Fully adjusted model**	**p value**
Healthy dietary pattern					
Low tertile	34	reference		Reference	
Middle tertile	26	0.70 (0.40-1.24)	0.224	0.69 (0.39-1.24)	0.213
High tertile	37	1.03 (0.57-1.84)	0.934	1.03 (0.57-1.88)	0.920
Western dietary pattern					
Low tertile	37	Reference		Reference	
Middle tertile	29	0.70 (0.41-1.20)	0.192	0.71 (0.41-1.21)	0.205
High tertile	31	0.71 (0.40-1.26)	0.237	0.71 (0.39-1.27)	0.246
Bread and confectionery dietary pattern					
Low tertile	31	Reference		Reference	
Middle tertile	34	1.09 (0.65-1.85)	0.738	1.08 (0.64-1.84)	0.771
High tertile	32	1.03 (0.59-1.77)	0.928	1.02 (0.59-1.78)	0.941
Alcohol and accompanying dietary pattern					
Low tertile	38	Reference		Reference	
Middle tertile	26	0.66 (0.39-1.13)	0.133	0.67 (0.39-1.15)	0.151
High tertile	33	0.93 (0.55-1.57)	0.775	0.94 (0.55-1.59)	0.807

The minimally adjusted model was adjusted for age, gender and exercise-habit, and the fully adjusted model was further adjusted for body mass index, amount of education, marital status, current smoking, history of hypertension and diabetes mellitus.

## Discussion

This cross-sectional study was designed to evaluate the relationship between dietary patterns and risk of depression in a community-dwelling Japanese population. We identified four dietary patterns. The healthy dietary pattern was characterised by a high intake of vegetables, seaweeds, tofu, fruits, and fish and was significantly correlated with the intake of vitamins B2, B6, B12, folate, and total n-3 PUFAs. Previous studies have suggested relationships between several B vitamins [25,26], n-3 PUFAs [7], and depressive symptoms. Therefore, we hypothesised that a dietary pattern containing high amounts of vegetables, fruits, and fish would be associated with a lower risk of depression [27,28]. However, no evident association was observed between any of the dietary patterns (including the healthy dietary pattern) and the risk of depression, regardless of the cumulative effects of possibly protective nutrients.

To date, some studies have shown a relationship between dietary patterns and depression among Western populations [13-15,29]. In a French study [13], a “healthy” cluster characterised by high fish intake in males and high fruits and vegetables intake in females showed a borderline significant association with fewer depressive symptoms in elderly females (beta = −0.16; 95% CI, -0.33 to 0.007). The males in the “pasta eaters” cluster had more depressive symptoms (beta = 0.26; 95% CI, 0.06 to 0.46). In a middle-aged British population [14], a “whole food” pattern characterised by high consumption of vegetables, fruits and fish was associated with a lower risk of depressive symptoms 5 years later, whereas a “processed food” pattern characterised by high consumption of sweetened desserts, fried foods, processed meats, refined grains and high-fat dairy products was associated with a higher risk. The SUN Project [29] examined a large Spanish cohort and found that stricter adherence to the Mediterranean dietary pattern (high intakes of vegetables, fruit and nuts, cereal, legumes and fish and a high ratio of monounsaturated- to saturated-fatty acids) was associated with a lower incidence of clinical depression. In addition, a “traditional” dietary pattern characterised by vegetables, fruit, meat, fish, and whole grains was associated with lower risk for major depression and dysthymia in Australian females [15].

Two studies of Japan, which has different dietary patterns than Western nations, have found contradictory results, however. Nanri and colleagues [16] found that a healthy Japanese dietary pattern characterised by high intakes of vegetables, fruit, mushrooms and soy products was associated with fewer depressive symptoms among municipal employees. By contrast, Okubo and colleagues [17] reported that neither the “Healthy” nor the “Japanese” pattern was related to the risk of postpartum depression. Only the first and second quartiles of the “Western” pattern, which had high loadings for vegetable oil, beef and pork, salt-containing seasonings, processed meat, chicken and eggs, had significantly different risks of postpartum depression; the second quartile had a lower risk, although no evident exposure-response associations were observed. One possible explanation for this inconsistency is that different background of study populations might affect our results. Lifestyle of municipal government employees and pregnant women might affect dietary patterns. Another explanation might be that age-related differences in dietary patterns affect our results. Our study population was relatively older than that of previous reports from Japan.

In addition, we cannot completely rule out an association between dietary patterns and depressive symptoms due to several limitations. First, the cross-sectional nature of the study does not allow for causal assumptions between dietary patterns and the onset of depressive symptoms. Future studies with longitudinal designs are needed to investigate these associations. Second, the depression diagnosis was established by the CES-D rather than by a clinician-administered structured diagnostic interview. Third, the dietary data were obtained using the BDHQ. Although the validity and reliability of our dietary questionnaire have been evaluated [20 ,23], potential misclassification of dietary patterns may have affected our results. Fourth, several potential confounding factors, such as employment physical activity levels and interpersonal relationships among families, were not assessed by our study. Employment physical activity may be a particularly important factor. High employment physical activity may have confounded the results. Stratification by employment physical activity should be a feature of future studies. Fifth, as all of the participants were volunteers who were interested in their health, they may have been healthier than the general population. Thus, the members of the community who were not in the study may have had differing depressive symptoms. This ‘selection bias’ must also be considered in studies of community populations. Finally, as our sample size was relatively small, we could not completely rule out beta error as the cause of our not detecting associations between dietary patterns and depression.

## Conclusions

This cross-sectional study of a Japanese community-dwelling population failed to find relationships between dietary patterns and risk of depression, regardless of the cumulative intake of possibly protective nutrients. Relatively small sample size, background of study populations, and older age of our participants could affect our discrepant results. In addition, the interpretation of our results was hampered by lack of data, including employment physical activity and longitudinal observations. Potential associations between dietary patterns and depressive symptoms were not completely ruled out. Future research exploring dietary patterns and depressive symptoms is warranted.

## Competing interests

The authors declare that they have no competing interests.

## Authors’ contributions

NS conceived the study, designed the study, conducted the statistical analysis, interpreted the data and wrote the initial draft of the manuscript. SK had full access to all of the data in the study and takes responsibility for the integrity of the data and the accuracy of the data analysis. NYF contributed to study design and assisted in drafting the manuscript. ST, AK, KT, KI, NO and IT participated in the data collection, and the interpretation of the results. All authors have approved the manuscript.
